# A genome-wide association study using Myanmar *indica* diversity panel reveals a significant genomic region associated with heading date in rice

**DOI:** 10.1270/jsbbs.23083

**Published:** 2024-12-04

**Authors:** Moe Moe Hlaing, Khin Thanda Win, Hideshi Yasui, Atsushi Yoshimura, Yoshiyuki Yamagata

**Affiliations:** 1 Plant Breeding Laboratory, Faculty of Agriculture, Kyushu University, 744 Motooka, Nishi-ku, Fukuoka 819-0395, Japan

**Keywords:** *indica* rice, heading date, GWAS, *PhyC*, haplotype

## Abstract

Heading date is a key agronomic trait for adapting rice varieties to different growing areas and crop seasons. The genetic mechanism of heading date in Myanmar rice accessions was investigated using a genome-wide association study (GWAS) in a 250-variety *indica* diversity panel collected from different geographical regions. Using the days to heading data collected in 2019 and 2020, a major genomic region associated with the heading date, designated as MTA3, was found on chromosome 3. The linkage disequilibrium block of the MTA3 contained the coding sequence (CDS) of the phytochrome gene *PhyC* but not in its promoter region. Haplotype analysis of the 2-kb promoter and gene regions of *PhyC* revealed the six haplotypes, PHYCHapA, B, C, D, E, and F. The most prominent haplotypes, PHYCHapA and PHYCHapC, had different CDS and were associated with late heading and early heading phenotypes in MIDP, respectively. The difference in CDS effects between the PHYCHapB, which has identical CDS to PHYCHapA, and PHYCHapC was validated by QTL analysis using an F_2_ population. The distribution of PHYCHapA in the southern coastal and delta regions and of PHYCHapC in the northern highlands appears to ensure heading at the appropriate time in each area under the local day-length conditions in Myanmar. The natural variation in *PhyC* would be a major determinant of heading date in Myanmar accessions.

## Introduction

Climate change is leading to potential droughts or floods. This threatens global crop production, especially for rain-fed crops that depend on unpredictable seasonal rainfall patterns. Rice is a staple food for half of the world’s population, with Asia contributing about 90% of global rice production (Schneider and Asch 2020). Rice production in South Asia is heavily relies on the monsoon season, during which more than 80% of the annual rainfall is received in most of the region (Bowden *et al.* 2023). In order to adapt to such a variable water availability due to climate change, it is critical to understand and appropriately adjust the heading date of rice varieties to ensure a duration suitable for rice production.

Myanmar is the seventh largest rice producer in the world, with 80% of its production consisting of rain-fed rice (Department of Agricultural Planning (DoP), Myanmar 2013). The typical periodic cycle of wet and dry seasons of the monsoon climate strongly influences seasonal and regional production. Heading date is a key agronomic trait for adapting rice varieties to different cultivation areas and crop seasons (Zhou *et al.* 2021). Rice cultivation takes place under a wide range of day length and temperature conditions, spanning from the cool northern highlands, to the central dry zone, and to the southern delta and coastal regions (Ministry of Agriculture, Livestock and Irrigation (MoALI), Myanmar 2015). Therefore, elucidating the genetic control of heading date in Myanmar rice varieties is crucial for adapting rice cultivation to different ecological zones and seasonal changes.

Genetic resources provide plant breeders with germplasm to develop new and improved cultivars with desirable characteristics. Rice landraces, especially ones from core collections established in gene banks, are important sources of valuable genes that can broaden narrow genetic bases and enrich existing varieties with favorable agronomic traits (Zeng *et al.* 2007). Although Myanmar is rich in terms of genetic diversity, research on Myanmar rice landraces is limited to information primarily accessible through isozyme analysis (Glaszmann 1987), SSR marker analysis (Saw *et al.* 2006, Thein *et al.* 2012, Wunna *et al.* 2016), and DArT techniques (Thant *et al.* 2021). The exploration of valuable genes from Myanmar rice landraces is essential to support breeding efforts in Myanmar and other rice-producing countries. However, studies on the genetic regulation of heading date in Myanmar rice landraces are not sufficiently archived.

Rice flowering time (hereafter, heading date) is regulated by several environmental and endogenous genetic factors (Wei *et al.* 2020). The regulation of rice flowering date involves a complex pathway controlled by several key genes. The rice *Heading date 1* (*Hd1*) gene, a homolog of *CONSTANTS* (*CO*) in Arabidopsis (Yano *et al.* 2000), promotes or represses heading under short day (SD) or long day (LD) conditions, respectively, by regulating the expression of the florigen gene *Hd3a* which is a mobile flowering signal in rice (Kojima *et al.* 2002, Tamaki *et al.* 2007). The *Early heading date 1* (*Ehd1*) gene in rice induces *FT-like* gene expression only under SD conditions and promotes flowering independently of *Hd1* (Doi *et al.* 2004). Another *FT-like* gene, *Rice Flowering Locus T 1* (*RFT1*), promotes floral transition under long-day LD conditions (Komiya *et al.* 2009, Ogiso-Tanaka *et al.* 2013). Two distinct flowering pathways have been reported in rice. Under SD conditions, flowering is regulated by the *OsGI-Hd1-Hd3a* (involving Arabidopsis *GI*, *CO* and *FT* ortholog genes) and *OsGI-Ehd1-Hd3a* pathways (Doi *et al.* 2004, Hayama *et al.* 2003, Kojima *et al.* 2002, Yano *et al.* 2000). Under LD conditions, the *OsGI-Ehd1-RFT1* pathway (Komiya *et al.* 2009) regulates rice flowering. Most day-length sensitive heading date QTLs, such as *Hd6*, *Ghd7*, *Hd17*, *DTH2*, and *Hd16*, function specifically under SD conditions (Hori *et al.* 2013, Matsubara *et al.* 2012, Takahashi *et al.* 2001, Wu *et al.* 2013, Xue *et al.* 2008). The heading date gene *Hd6*, identified in the progeny of a backcrossed progenies of between the *japonica* rice variety Nipponbare and in the *indica* rice variety Kasalath, has been associated with photoperiod sensitivity (Takahashi *et al.* 2001). The rice heading date gene located on chromosome 8, *Heading date 5* (*Hd5*)/*Grain number, plant height, and heading date8* (*Ghd8*)/*days to heading 8* (*DTH8*) genes, was associated with delayed heading under LD conditions (Lin *et al.* 2003, Wei *et al.* 2010, Yan *et al.* 2011). Loss of function of *Hd5* results in an extremely early heading which is considered to be an adaptation characteristic of the northern limits of rice cultivation (Fujino *et al.* 2013). In addition, phytochromes are important regulators for photoperiod flowering in rice (Izawa *et al.* 2000). Arabidopsis contains five phytochrome genes, *phyA* to *phyE*, while rice has only three, namely *phyA*, *phyB*, and *phyC* (Basu *et al.* 2000). Using the mutant lines of rice, the mutation in *phyB* or *phyC* showed moderate early flowering in long-day conditions, while the *phyA* in combination with *phyB* or *phyC* showed early flowering in rice (Takano *et al.* 2005).

Agronomic QTL mapping involves the use of populations such as F_2_ and recombinant inbred lines (RILs) (Takeda and Matsuoka 2008). Next-generation sequencing (NGS) technologies, when combined with precise phenotyping methods, offer the potential to elucidate the genetic basis of important agricultural traits (Varshney *et al.* 2014). Genome-wide association study (GWAS) consists of a statistical method that evaluates the association between nucleotide polymorphism and phenotypic variation and is a powerful tool for identifying QTL associated with specific traits (Uffelmann *et al.* 2021). A study revealed the genetic basis of 14 agronomic traits using 517 *indica* rice landraces demonstrating that GWAS is a powerful complementary approach to classical biparental cross-mapping for exploring complex traits in rice (Huang *et al.* 2012b). Furthermore, the mixed model approach is the first choice of plant scientists for controlling false positive associations in population mapping studies (Myles *et al.* 2009).

Recently, a Myanmar *indica* diversity panel (MIDP), comprising a set of 250 *indica* rice landrace accessions and genome-wide nucleotide variants obtained by whole genome sequencing, was developed to facilitate molecular genetic studies on untapped Myanmar rice germplasm (Furuta *et al.* 2024). These resources can provide the means to elucidate the genetic basis underlying heading date in the Myanmar rice landraces and to develop novel varieties in rice with improved regional and seasonal adaptability.

In this study, we conducted a GWAS to investigate the genetic basis of heading date using the MIDP. The genomic region associated with the heading date in Myanmar rice landraces was elucidated and the haplotypes of the candidate gene, *PhyC*, were explored from their natural variation. Our study reveals that the two *PhyC* haplotypes were associated with late and early heading and are likely to play an important role in adapting rice to local day length conditions and ensuring timely heading in different regions.

## Materials and Methods

### Plant material

**Fig. 1.**
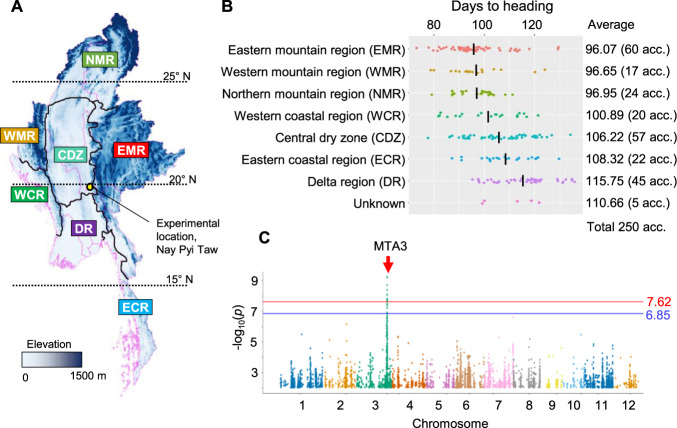
Phenotypic variation of days to heading (DTH) in 250 Myanmar *indica* rice accessions. (A) Locations of geographical regions. (B) DTH by region and their average. The number in the parenthesis represents number of accessions. (C) Manhattan plot for GWAS of average DTH in 2019 and 2020. The blue and red horizontal lines represent thresholds at 5% significant level determined by Bonferroni correction and 100 times permutation test. Only one significant peak of marker-trait association designated MTA3 was found on chromosome 3.

The MIDP (Furuta *et al.* 2024), consisting of 250 *indica* accessions collected from different geographical areas and preserved in the Seed Bank, Department of Agricultural Research (DAR), Nay Pyi Taw, Myanmar, was used in the GWAS. The accessions of the diversity panel were classified according to their geographical origin: the central dry zone (CDZ), delta region (DR), eastern coastal region (ECR), eastern mountainous region (EMR), northern mountainous region (NMR), western coastal region (WCR), and western mountainous region (WMR) (Fig. 1A). Map data of the administrative areas (boundaries) and elevation in Myanmar were downloaded from https://www.diva-gis.org/gdata and plotted using QGIS software (https://www.qgis.org). The experimental field was located in the Rice Research Section, DAR, Nay Pyi Taw, Myanmar (19.82° N and 96.26° E) (Fig. 1A). Conventional field management practices were used during the experiment. Forty seeds of each accession were germinated, and seedlings were transplanted into the experimental field in two rows with 24 individuals using a spacing of 25 cm between plants and 25 cm between rows. For both 2019 and 2020, the sowing date of the MIDP was July 6th, and the transplanting date was July 30th.

The F_2_ population for the QTL analysis was derived from a cross between the MIDP accession ‘Kauk Hynin’ (accession CC319) as the female parent and the Vietnamese improved variety ‘KhangDan18’ (KD18) as the male parent. KD18 was used as a polymorphic parent to obtain more frequent polymorphic SNPs in genotyping-by-sequencing. A total of 118 F_2_ individuals were used for the QTL analysis.

### Evaluation of days to heading

Days to heading (DTH) were evaluated every two days by recording the number of days from sowing to the first panicle emerging from the flag leaf sheath (2 cm above the panicle). The experiments were conducted during two different sowing seasons: the 2019 and 2020 monsoon seasons. The average DTH of eight randomly selected individuals in each accession was evaluated. During the monsoon season, the average day length in the Nay Pyi Taw area is 12.0–13.55 hours, and the average temperature is around 25–30°C over two years.

### Whole genome sequencing and SNP identification

Whole genome sequencing and variant calling analysis were conducted according to Furuta *et al.* (2024). Genomic DNA was extracted using the potassium acetate method (Dellaporta *et al.* 1983) with minor modifications. Whole genome sequences of MIDP were determined using the DNA nanoball-sequencing (DNB-seq) technology at 12.3 times the total depth of the Nipponbare reference genome (420 Mb in a haploid genome). Low-quality score bases were eliminated using the Trimmomatic software (Bolger *et al.* 2014) by applying the parameters ILLUMINACLIP:TruSeq3PE.fa:2:30:10:2:keep- BothReads LEADING:3 TRAILING:3 SLIDINGWINDOW:4:15 MINLEN:3. The qualified pair-end reads were mapped based on the Nipponbare reference sequence (Os-Nipponbare-Reference-IRGSP-1.0, Kawahara *et al.* 2013) using the bwa-mem software (https://arxiv.org/abs/1303.3997). The variant calling procedure followed the best practice workflow of the germline short variant discovery in the Genome Analysis Tool Kit version 4.1.3 (GATK4) software (https://www.biorxiv.org/content/10.1101/201178v3). The heterozygous genotypes were converted to the missing genotypes by the custom Perl script. Variants were filtered using the vcftools software (Danecek *et al.* 2011) with minor allele frequencies (MAF) of more than 0.05 and genotype missing frequencies of less than 0.1. Genotype imputation was performed before running permutation-based GWAS using Beagle5.4 software (Browning *et al.* 2018), and 2,079,574 SNPs were retained for the GWAS.

### Genome-wide association

Permutation-based GWAS of DTH was performed using the R package permGWAS2 (John *et al.* 2022. The fixed effect of SNP genotypes and the random effect of genetic background (kinship) were simultaneously estimated using the mixed linear model (MLM) in the permGWAS2 library. The genome-wide threshold was determined by Bonferroni correction and 100 times permutation test at a 5% significant level, according to John *et al.* (2022). Mixed linlear model GWAS without genotype imputation was performed using the R package rrBLUP (Endelman 2011). The estimated genome realized matrix was obtained using the A.mat() function. Quantile-quantile (QQ) plots were drawn using the R/qqman library (Turner 2018).

### Linkage disequilibrium and haplotype analysis

Local linkage disequilibrium (LD) of the pairwise SNPs was visualized using the R/LDheatmap package (Shin *et al.* 2006) around the significant peak regions. Boundary SNPs of the LD block were confirmed by visual observation in the Tassel-GUI (Bradbury *et al.* 2007). For haplotype analysis in candidate gene, the raw and unfiltered genotype data were used to examine possible variants of the SNPs and Indels. After omitting missing data and heterozygous, the SNPs and Indels in the coding region and 2 kb promoter region were analyzed for haplotype analysis. Tukey’s honestly significant difference (HSD) test was performed to test significant difference of each haplotype. No significant differences were observed among haplotypes that shared the same letter designation. The haplotypes were classified based on the minor allele frequency 0.03, at least seven accessions were considered for the one haplotype group.

### Variant annotation

The functional effects of the variants on genes were predicted using SnpEff version 4.3T (Cingolani *et al.* 2012). The default dataset ‘Oryza sativa’ for the Os-Nipponbare-Reference-IRGSP-1.0 reference sequence was employed for the analysis. Single nucleotide and insertion/deletion variant VCF files with a missing rate of less than 10% and a minor allele frequency (MAF) of at least 0.05 were used as input for annotation.

### Genotyping-by-sequencing and genotype call

For genotyping in the F_2_ population, genomic DNA was extracted from fresh leaf samples using the method as described above. Library preparation adapted to the Illumina dual index system was performed according to Poland *et al.* (2012) with minor modifications. Briefly, genomic DNA samples were digested with *Pst*I and *Msp*I restriction enzymes at 37°C for 2 hours, followed by an inactivation step at 65°C for 15 minutes. An oligonucleotide pair of the adaptor compatible with the *Pst*I sticky end, 5ʹ-CACGACGCTCTTCCGATCT (barcode sequence) TGCA-3ʹ and 5ʹ-(complementary barcode sequence) AGATCGGAAGAGCGTCGTG-3ʹ and another pair compatible with the *Msp*I end, 5ʹ-CGAGATCGGAAGAGCACACTCTTTCCCTACACGAC-3ʹ and 5ʹ-GTGACTGGAGTTCAGACGTGTGCTCTTCCGATCT-3ʹ, were annealed in AB buffer. After an adaptor ligation reaction containing a set of 96 unique barcodes and pooling samples, the obtained product was purified by Qiagen QIAquick PCR Purification Kit (Qiagen, Venlo, The Netherlands) and amplified using NEBNext i5 and i7 primers (New England Biolabs, Ipswich, MA, USA) compatible with the Illumina dual index system using KAPA HiFi HotStart ReadyMix (Kapa Biosystems, Wilmington, MA, USA). DNA fragments of 200–700 bp were obtained using 0.85–0.56 × SPRIselect beads (Beckman Coulter, Brea, CA, USA). Following library quantitation by quantitative PCR, prepared libraries were sequenced using Illumina NovaSeq 150 PE platform (Azenta, Tokyo, Japan).

The resulting fastq files were analyzed using the TASSEL GBS V2 pipeline (Glaubitz *et al.* 2014). The LB-Impute software was used to impute missing genotypes (Fragoso *et al.* 2016). Between the parents, the polymorphic SNP genotypes were selected and converted to ABH genotypes in the custom script (https://github.com/qikushu/gbs). QTL analysis was performed with marker regression using the scanone() function in the R/qtl library (Broman *et al.* 2003). The threshold of the logarithm of odds (LOD) was determined using the top 5% level of the empirical distribution of LOD in a 1,000 times permutation test.

### Genotyping using InDel markers

To develop agarose-gel-based indel markers around the QTL region detected by the GBS and genotype imputation, whole genome variant information of the CC319 and KD18 was combined with the GATK4/CombineGVCFs command, and their indel variants were called with GATK4/GenotypeGVCFs. Indel variants were screened using SelectVariants with the ‘-select-type INDEL’ option. A total of three InDel markers covering the MTA3 were designed (Supplemental Table 1). The PCR reaction contained an amount of approximately 10 ng of genomic DNA, 0.2 M primers, and 7.5 μl of GoTaq Master Mix (Promega, Fitchburg, WI, USA). The thermal profile included an initial denaturation at 95°C for 5 min, 35 cycles of 95°C for 30 s, 55°C for 30 s, and 72°C for 30 s, and a final elongation step at 72°C for 7 mins. The amplified products were visualized by a 4% agarose gel electrophoresis in 0.5× TBE buffer.

## Results

### Phenotypic variation in days to heading among Myanmar accessions

DTH of the Myanmar diversity panel was investigated during the 2019 and 2020 monsoon seasons (Supplemental Fig. 1A, 1B, Supplemental Table 2). Given that two-year replicates were sown on the same day each year, it is assumed that they were exposed to nearly identical day-length conditions each year despite varying climate conditions. High correlations (*r* = 0.94) observed in the DTH across the two years were attributed to the day-length conditions. To estimate a robust heading date for the varieties unaffected by the differences in weather conditions across multiple years, the mean value of DTH over the two years was also calculated for subsequent studies. The MIDP exhibited DTH values ranging from 73.0 to 139.5, with an overall mean of 104.2 days (Fig. 1A, 1B, Supplemental Fig. 1A, 1B, Supplemental Table 2). These suggest that landraces of the diversity panel have high genetic diversity in terms of DTH. Accessions from the northern highlands, including EMR, WMR, and NMR, exhibited earlier heading dates with mean DTH values of less than 100 days (Fig. 1B). Conversely, those from the southern coastal and delta region such as WCR, ECR, and DR, showed later heading dates (Fig. 1B). The accessions from the central region (CDZ) showed a broad range of DTH variation and had intermediate mean DTH values. The correlation coefficient between the latitude and the DTH for the MIDP accessions was –0.35, implying that the genetic control of heading date in MIDP may be associated with latitude, likely due to adaptations to local day length variations.

### Genome-wide association analysis

To explore genomic regions associated with the DTH in the MIDP, permutation-based GWAS was conducted not only for each of the monsoon seasons of 2019 and 2020 but also using the mean values of two years (Supplemental Fig. 2, Fig. 1C, Table 1). The peak SNPs identified in each GWAS were located within a single linkage disequilibrium (LD) block, spanning approximately between 31.0 and 31.31 Mb (Table 1). The highest peak SNPs in the MTA3 region were found at 31,115,122 bp, 31,115,130 bp, and 31,115,163 bp based on the GWAS utilizing DTH data from 2020. In contrast, using data from 2019 and the average over the two years, the highest peak SNP in the MTA3 region was at 31,063,052 bp. In the two-year average data, accessions with the G nucleotide at 31,063,052 bp of the MTA3 peak SNP showed a DTH of 95.55, whereas those with the A nucleotide headed approximately 18 days later, with a DTH of 113.53, in the diversity panel (Table 1).

Using variant data without imputation, a GWAS was conducted using the rrBLUP software (Supplemental Fig. 3). A significant peak on chromosome 3 was located at 31,113,473 bp for the heading date in 2019, 2020, and their average GWAS within the same LD block of MTA3, suggesting that genotype imputation did not affect the detection of MTA3 (Supplemental Table 3). On chromosome 6, a peak was detected by rrBLUP that was not detected by permGWAS2. There are no previously reported isolated QTLs around this peak, indicating the potential presence of an unknown QTL on chromosome 6 in this panel.

There were 47 annotated genes included in the LD region of the MTA3 (Supplemental Table 4, Supplemental Fig. 4). Gene models in the MTA3 region were classified into four candidate classes (A–D). Class A is defined as “genes identical to the locus known to be related to reproductive transition”, class B as “genes sharing homologous regions with genes related to days to heading”, class C as “annotated genes”, and class D as “hypothetical genes without annotated structural features”. Three genes classified into the class A were *Os03g0752100* (*PhyC*), *Os03g0752800* (*OsMADS14*), and *Os03g0753100* (*OsMADS34*) (Supplemental Fig. 4B). Five SNP in exon, five SNP in intron, and one SNP in 3ʹ UTR of the *PhyC* were associated with DTH with a –log10(*p*) value greater than 6.5. Two SNP in the intron of *OsMADS14* have –log_10_(*p*) values of 6.52 and 5.29, respectively (Supplemental Table 5). *OsMADS34* is not associated with the heading date phenotype, a SNP found in exon3 of *OsMADS34* has a –log_10_(*p*) of 0.6 (Supplemental Table 6). These results indicated that *PhyC* is the most likely candidate gene for the MTA3 peak.

The seven class-B genes in the MTA3 region, *Os03g0754900*, *Os03g0755000*, *Os03g0756200*, *Os03g0757100*, *Os03g0757200*, *Os03g0757500*, and *Os03g0757600*, showed partial homologies to deduced protein data sets derived from the 195 genes with the trait ontology “days to heading” (TO:0000137) in the RAP-DB database (Supplemental Table 7) in the BLASTp searches (Altschul *et al.* 1990) with a threshold of evalue 0.01. The 18 class-C genes had deduced functional descriptions in the RAP-DB, but the other 19 class-D genes were hypothetical, and their functions are still unknown (Supplemental Table 8). Expression profiles of the candidate genes in the flag leaves before and after heading (Wang *et al.* 2013) in the RAP-DB database showed that all genes, except the four class-D genes, *Os03g0752450*, *Os03g0753400*, *Os03g0753750*, and *Os03g0757051*, were expressed at detectable levels in the RNA-seq at both stages (Supplemental Table 7). Structural variats leading the protein sequence changes were identified by snpEff program and the variants with the HIGH, MODERATE, and LOW impact flags were integrated with the –log_10_(*p*) values in GWAS to clarify their associations to the days-to-heading (Supplemental Table 8). The sequence variants found in *Os03g0752200* (class C), *Os03g0755100* (class C), *Os03g0755600* (class C), *Os03g0757000* (class C), *Os03g0757750* (class D) exceed over the threshold level 6.85 in GWAS. These loci, following *PhyC*, may represent previously unknown loci governing heading.

### Haplotypes at *PhyC*

**Fig. 2.**
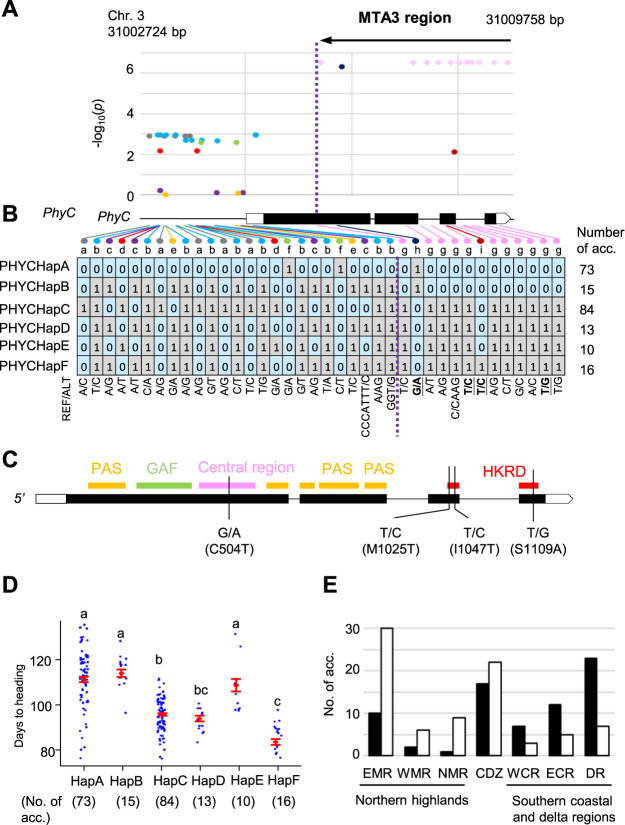
Haplotypes of *PhyC*. (A) Local Manhattan plot on coding region and 2 kb promoter region of *PhyC*. SNP and indel polymorphisms around *PhyC* region in the MIDP are shown in different colors according to nine segregation patterns (See Supplemental Fig. 4B). The violet vertical line indicates the 5ʹ boundary of the LD block of MTA3. (B) Blue and gray blocks represent the reference and alternative alleles of each SNP, respectively, where ‘0’ indicates the reference allele identical to Nipponbare and ‘1’ represents the alternative allele. A recombination hotspot was found between the 5ʹ UTR and the first exon of *PhyC*. (C) Domain structure of PhyC shown on gene structure. Locations of the period–ARNT–single-minded (PAS) domain, cGMP-specific phosphodiesterase–adenylyl cyclase–FhlA (GAF) domain, central region domain, and histidine kinase-related domain (HKRD) are represented in yellow, green, pink, and red bars, respectively. One and three onsynonymous polymophism were found in the central region domain and HKRD. (D) Average DTH by the haplotypes. The haplotypes marked with the same letter did not show significant differences at the 5% level in Tukey’s honestly significant difference test. (E) Haplotype frequency of the predominant haplotypes of *PhyC* by the seven geographical regions. Black and white bars represent the frequencies of PHYCHapA and PHYCHapC, respectively.

The haplotypes within the coding region (CDS) and 2 kb promoter region of *PhyC* were investigated (Fig. 2, Supplemental Table 9). A local Manhattan plot was drawn at the six SNPs in the CDS, five SNPs and one indel polymorphism in the intron region, two indel polymorphisms in the 5ʹ untranslated region (UTR), and one SNP in the 3ʹ UTR of the *PhyC* gene region (Fig. 2A). The CDS regions contained two synonymous and four nonsynonymous polymorphisms. In addition, twenty-three variant sites, including SNP and indels, were found in the 2 kb promoter region. These polymorphisms across the gene and promoter regions of *PhyC* revealed the six distinct haplotypes, designated as PHYCHapA, B, C, D, E, and F (Fig. 2B). The PHYCHapA and PHYCHapC haplotypes were found to be the most predominant in the MIDP.

In this genomic region, the nine segregation patterns of the variants (a, b, c, d, e, f, and g) were observed (Supplemental Fig. 5).The –log_10_(*p*) values of the twelve SNPs and one indel polymorphism that showed the segregation pattern g, h, and i, in the CDS, intron and 3ʹ UTR of *PhyC* were relatively high because they were located within the LD block of the MTA3 (Supplemental Fig. 5A). However, the polymorphisms harboring with the segregation patterns a, b, c, d, e, and f in the promoter region and 5ʹ UTR were outside of the MTA3, and their –log_10_(*p*) values were suddenly decreased (Supplemental Fig. 5A). It is likely that recombination hotspots exist between the SNPs at 31,006,150 (CDS) and 31,004,922 (5ʹ UTR) bp in the Nipponbare reference sequence within the MIDP. These data suggest that polymorphisms in the coding sequences of *PhyC*, or in genomic regions downstream of *PhyC*, are associated with DTH in the MIDP.

In the CDS of *PhyC*, a nonsynonymous SNPs G/A leading to a cysteine and threonine polymorphism was found at the 504th amino acid residue within the central region domain of the PhyC protein which was designated [G/A (C504T)] (Fig. 2C). Similarly, in the histidine kinase-related domain (HKRD), three nonsynonymous SNPs were identified; T/C (M1025T), T/C (I1047T), and T/G (S1109A). The T/C (I1047T) nonsynonymous polymorphism with the segregation pattern i showed low association, whereas the other nonsynonymous polymorphism with the segregation pattern g showed high association with DTH (Fig. 2C).

The genetic effects of the six haplotypes were examined by multiple comparison using Tukey’s HSD test (Fig. 2D). This test showed that the haplotypes PHYCHapA, PHYCHapB and PHYCHapE belonged to the same significant group, which delayed heading than the other haplotypes PHYCHapC, PHYCHapD, and PHYCHapF. PHYCHapA and PHYCHapB have identical CDS but differ in their promoter and 5ʹ UTR sequences. These suggest that the genetic effects of PHYCHapA and PHYCHapB on DTH could not be distinguished.

### Validation of QTL using GBS in F_2_ population

**Fig. 3.**
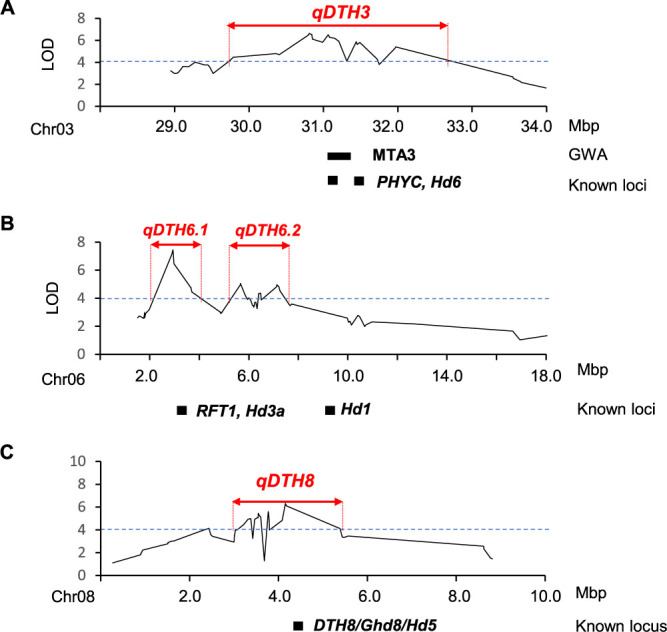
The QTL controlling DTH detected in biparental mapping population derived from crossed between CC319 and KD18. The LOD curves of (A) QTL detected on chromosome 3 (B) QTLs detected on chromosome 6 and (C) QTLs detected on chromosome 8. The horizontal blue bar represented LOD threshold (5% level of significant). The vertical red bar represented the genomic region of each QTL. The horizontal black bar represented the MTA detected from GWAS and position of known loci.

The genetic effects of the most predominant haplotypes of *PhyC* in the MIDP, PHYCHapA and PHYCHapC, were investigated by QTL analysis using an F_2_ population derived from a cross between the accession CC319, which carries PHYCHapA, and the *indica* variety KD18 from Vietnam, which carries PHYCHapC (Fig. 3). A total of 3,663 GBS markers covering twelve rice chromosomes and the three Indel markers around MTA3 were used for marker regression in single marker analysis.

Four significant QTLs were detected on chromosomes 3, 6, and 8 in the F_2_ population (Table 2, Fig. 3, Supplemental Fig. 6). The significant effect QTL detected on chromosome 3, designated as *qDTH3*, harbours the peak SNP at 30,809,923 bp with a LOD value of 6.39 and explaining 22.08% of the phenotypic variance explained (PVE). The genomic region with an LOD value above the threshold was located between the 30,351,246 and 31,694,280 bp regions. This QTL region contained the *PhyC* and *Hd6* regions on the Nipponbare reference sequence (Fig. 3A).

Two other major QTL, *qDTH6.1* and *qDTH6.2*, are located between 2,940,130 and 3,563,230 bp and between 5,660,320 and 7,843,157 bp, respectively, on chromosome 6 (Table 2, Fig. 3B). *qDTH6.1* and *qDTH6.2* exhibited peak LOD scores of 6.29 and 4.81, respectively. Notably, the *qDTH6.1* region encompasses two key florigen genes: *Hd3a* (chr06: 2,940,004–2,942,452 bp) and *RFT1* (chr06: 2,926,823–2,928,474 bp). In contrast, the *qDTH6.2* region does not contain any specific genes directly linked to the heading date. However, it is situated very close to the important heading date genes, *Hd3a* and *Hd1* (chr06: 9,336,376–9,338,569 bp) (Fig. 3B).

The QTL, designated as *qDTH8*, was detected from 3,317,965 to 5,572,424 bp on chromosome 8 of the Nipponbare genome. This QTL recorded the highest LOD score of 8.09 and had the highest PVE of 27.08% among the four detected QTLs. A major heading date gene, *Hd5/DTH8/Ghd8*, was found in the same location as *qDTH8*. (Table 2, Fig. 3C).

Multiple QTL mapping was performed including the genetic effect at *qDTH3*, *qDTH6.1*, *qDTH6.2*, or *qDTH8*. However, no additional QTL was detected (data not shown).

### Geographical distribution of the predominant haplotypes of *PhyC*

The frequencies of the predominant *PhyC* haplotypes, PHYCHapA and PHYCHapC, were examined across seven geographical regions in Myanmar (Fig. 2E, Table 3). Compared to PHYCHapC, PHYCHapA was found to have a lower frequency in the northern highlands including EMR, NMR, and WMR, and a higher frequency in the southern coastal and delta regions such as ECR, WCR, and DR (Fig. 2E). This was examined through testing the goodness of fit to the population haplotype frequencies in a ratio of 73:84 for the PHYCHapA and PHYCHapC at the 5% significance level (Table 3). These distributions appear to ensure heading at the appropriate time in each area under the local day length conditions in Myanmar.

## Discussion

### Major genetic factors regulating heading date variation in Myanmar

To elucidate the genetic mechanism controlling heading date in Myanmar rice landraces, GWAS for days to heading (DTH) using the MIDP was performed. Only one significant marker-trait association was found in the single LD block, designated as MTA3 (Table 1, Supplemental Fig. 2). The MTA3 region contains the 47 annotated genes on the Os-Nipponbare-Reference-IRGSP-1.0. Three, seven, eightteen, and nineteen genes were suggested to classify into the four candidate classes, class A–D, respectively, according to the identities or similarities to the genes conferring heading or reproductive transitions (Supplemental Table 4). The class-A genes, *PhyC*, *OsMADS14* and *OsMADS34*, were located in the upstream region of MTA3 peak about 53 kb, 31 kb and 15 kb, respectively (Supplemental Fig. 4). The mutation in *PhyC* caused early flowering under LD conditions (Takano *et al.* 2005). A *phyC* mutant, *late heading date 3* (*lhd3*) mutant exhibited delayed flowering under both long-day and short-day conditions, and delayed flowering time was positively associated with the day length via the *Ehd1* pathway (Lin *et al.* 2022). In rice, *PANICLE PERFORMER 2* (*MADS34*) and the three *APETALA1 (AP1)/FRUITFULL (FUL)-like* genes (*MADS14*, *MADS15*, and *MADS18*) act coordinately in the meristem to specify the identity of the inflorescence meristem downstream of the florigen signal (Kobayashi *et al.* 2012).

The class-B gene *Os03g0754900/OsWD40–93* showed homologies to the genes related to days to heading *Os02g0771100/PETER PAN SYNDROME* (*PPS*) (Tanaka *et al.* 2011) and *Os03g0725400/OsWDR5a/OsWD40–86* (Jiang *et al.* 2018) via WD40 domains (Supplemental Table 10). Although 234 putative *OsWD40* genes has been identified (Ouyang *et al.* 2012), not all genes with WD40 domain control days to heading. Similarly, another class-B genes *Os03g0755000* and *Os03g0756200* shared the protein kinase domain (IPR000719) with *Os02g0527600/CONSTITUTIVE TRIPLE-RESPONSE2* (*CTR2*) (Wang *et al.* 2013), *Os03g0793500/HEADING DATE 16* (*HD16*) (Hori *et al.* 2013), and *Os08g0484600/PROTEIN KINASE 4* (*OSK4*) (Sun *et al.* 2016). The class-B gene cluster including *Os03g0757100*, *Os03g0757200*, *Os03g0757500*, and *Os03g0757600* all showed homology to *Os04g0206700*/*UGT74J1* (Tezuka *et al.* 2021). But only sharing functional domains would not be sufficient evidence for regulation of days to heading by these genes.

When focusing on nucleotide variants associated with structural variations in proteins, the five candidate genes, *Os03g0752200* (class C), *Os03g0755100* (class C), *Os03g0755600* (class C), *Os03g0757000* (class C), and *Os03g0757750* (class D) involved the variants highly associated with days to heading in GWAS (Supplemental Table 8). Disruptive mutations at *Os03g0752200*, identical to *OsSPO11*, induced absence of bivalent formation at metaphase I and male meiosis deficiency (An *et al.* 2011, Fayos *et al.* 2020). *Os03g0755100* (*OsALS1*), encodes a half-size ABC transporter and their knockout lines resulted in significant increased sensitivity to aluminum (Huang *et al.* 2012a). *Os03g0755600* is found to have a HAD superfamily domain (IPR023214), but biological function of this gene has not been fully understood. *Os03g0757000* was annotated as UDP-glucuronosyl/UDP-glucosyltransferase domain containing protein and showed partial sequence homology to *Os04g0206700* encoding UDP-glucosyltransferase 74J1 (UGT74JI). *UGT74JI* is expressed ubiquitously throughout plant development and controls basal salicylic acid (SA) levels. The *ugt74ji* mutants generated by genome editing showed high level accumulation of SA under non-stressed conditions, reduced plant stature, and delayed heading (Tezuka *et al.* 2021). Other than *PhyC*, these genes may partially contribute to the variation in days to heading in MIDP.

We then analysed the variation of DTH in haplotypes of *PhyC* in MIDP. Multiple comparisons on the six haplotypes suggested that the PHYCHapA, PHYCHapB, and PHYCHapE haplotypes had the genetic function resulting in late heading compared to PHYCHapC, PHYCHapD, and PHYCHapF haplotypes. Despite having an identical CDS, PHYCHapA and PHYCHapB differ in their promoter and 5ʹ UTR sequences, suggesting that their genetic effects on DTH could not be distinguished. The PHYCHapA haplotype is predominant in the southern coastal and delta regions such as WCR, ECR, and DR. In contrast, the HapC haplotypes are more common in the northern highlands including EMR, WMR, and NMR (Fig. 2E, Table 3). The different genetic effects in DTH between the PHYCHapB and PHYCHapC were confirmed in the conventional QTL mapping in F_2_ populations (Table 2, Fig. 3). The phytochrome gene, *PhyC*, was proposed to be a major determinant of the heading date in the Myanmar accessions.

GWAS revealed that MTA3 is located in the 53-kb downstream region of the phytochrome gene *PhyC* (Supplemental Fig. 4A). The local Manhattan plot at *PhyC* showed that the CDS in *PhyC* was highly associated to the DTH (Fig. 2A–2C). The long and intronless 5ʹ UTR of the *PhyC* transcript is a unique feature of rice *PhyC* gene as compared to other *PhyC* family members, which may have functional significance to *PhyC* expression or activity (Basu *et al.* 2000). We found one polymorphism in the central region and three polymorphisms, T/C (M1025T), T/C (I1047T), and T/G (S1109A), in the histidine kinase-related domain (HKRD) (Fig. 2C). The biological function of the HKRD seems to be dimerization and an ATP/ADP-binding phosphotransfer or catalytic domain (Rockwell *et al.* 2006). Of the three polymorphisms in the HKRD, T/C (M1025T) and T/G (S1109A) were corresponded to the conserved residue associated with the ATP catalytic pockets in *phyA* in *Arabidopsis* (data not shown). Further studies are needed to understand how the amino acid substitutions introduced by these SNPs affect heading in rice.

The major heading date gene *Hd6* was located in another LD immediately adjacent to the LD block containing the MTA3 peak and 440 kb away from MTA3 peak (Supplemental Fig. 4). There is another possibility that MTA3 might be a misleading association of *Hd6*. Misleading associations are caused by SNPs pseudo-positive signals as demonstrated in a *Hd1* GWAS using a set of 176 japonica rice accessions (Yano *et al.* 2016). The 43-bp deletion in the coding sequence of *Hd1* kept high LD with SNPs located approximately 1 Mbp upstream of *Hd1*. The significant region of *qDTH3* in the F_2_ population derived from a cross between CC319 and KD18 contained *Hd6*. However, the potential causal polymorphism was not found in the variants in this study (data not shown), although it showed high LD (Supplemental Fig. 4C).

The geographical distribution of the predominant haplotypes, PHYCHapA and PHYCHapC (Table 3), suggest that *PhyC* is associated with the distribution of varieties along latitudes. The PHYCHapA haplotype was frequently observed in late-heading varieties. The varieties possessing the PHYCHapA haplotype originated from the coastal and delta regions (Fig. 2E). In water logging or flood-prone areas including the coastal (ECR and WCR) and delta (DR) regions, it can be assumed that a delayed flowering is advantageous to avoid submergence of headed panicle in water and excessive yield loss during the monsoon season. On the other hand, varieties with early heading were observed in the PHYCHapC haplotype. These varieties were frequently distributed in areas with low annual rainfall, including the central dry zone (CDZ) and mountainous regions (EMR, NMR, and WMR) (Fig. 2E), probably because the early transition to the reproductive stage ensures sufficient levels of water and temperature for maturation before the coming of the dry season and low temperatures in highlands. The average temperature in December ranged from about 13.9°C and 25.6°C during 1991–2020 at the NMR (Kachin state) and ECR (Tanintharyi division), respectively (https://climateknowledgeportal.worldbank.org). The geographical distribution of the haplotype on heading-related QTL would play an important role in addressing environmental challenges related to adaptability.

### Other genetic factors regulating heading date

Conventional QTL analysis in the F_2_ population revealed three genomic QTL regions (*qDTH6.1*, *qDTH6.2*, and *qDTH8*) (Table 2, Fig. 3B, 3C) that were not detected in GWAS (Table 1, Fig. 3B, 3C). The F_2_ populations were generated by crossing the Myanmar rice varieties CC319 with the Vietnamese variety KD18. Therefore, it is likely that certain alleles at the loci might be fixed in the MIDP, while the KD18 alleles may be functionally different from those in MIDP. The important florigen genes, *Hd3a* and *RFT1*, were included in the genomic region of *qDTH6.1*, which was detected in the F_2_ population on chromosome 6 (Fig. 3B). Similarly, the *qDTH8* region contained *Hd5/DTH8/Ghd8* genes as revealed by the F_2_ analysis (Fig. 3C). It is reported that polymorphism at *Hd5* has been associated with extremely early heading and adaptation to the northern limit of rice cultivation in Hokkaido, Japan (Fujino *et al.* 2013). The PHYCHapA haplotypes might be necessary for late heading in lower latitude areas like Myanmar.

The allelic variation in the *Hd1* and *Ghd7* genes provides a wide range of adaptability in rice growing areas of Asia (Takahashi *et al.* 2009, Xue *et al.* 2008). However, the major heading date related genes, *Hd1* and *Ghd7*, were not detected in either GWAS or F_2_ analysis in this study. The functional *Ghd7* alleles were well adapted at latitudes lower than 35°N in the tropics and subtropics, while the non-functional alleles were adapted in latitudes higher than 45°N (Xue *et al.* 2008). The functional alleles of *Ghd7*, *Ghd8*, and *Hd1* combinations were highly sensitive to the photoperiod and are best suited for cultivation in tropical areas where the day length is short, and the temperature is warm all year round (Zhang *et al.* 2015). *Hd6* has been found to inhibit flowering under LD conditions and requires *Hd1* to express its flowering-inhibitory function (Ogiso *et al.* 2010). It can be speculated that most of the Myanmar accessions carry functional *Hd1* and *Ghd7* alleles.

In conclusion, the present study clearly revealed the major genetic factor controlling heading date in Myanmar *indica* accessions by GWAS and conventional QTL analysis. The allelic variation of the candidate gene, *PhyC*, provides useful information on favorable haplotype parental selection. This research can be used as a basis for future studies to explore useful genes associated with both in agronomically important traits and biotic and abiotic stress tolerance traits. It also provides insights into the genetic control of heading date in Myanmar rice varieties which is crucial for their adaptation to different ecological zones and seasonal changes.

## Author Contribution Statement

A.Y. and Y.Y. conceptualized this study. M.M.H. performed overall research work. K.T.W. and A.Y. performed plant cultivation and phenotyping in F_2_. A.Y. and H.Y. provided the seeds. The manuscript was initially written by M.M.H. and reviewed and edited by M.M.H. and Y.Y..

## Supplementary Material

Supplemental Figures

Supplemental Tables

## Figures and Tables

**Table 1.** Significant SNPs associated with days to heading detected in Myanmar *indica* diversity panel

Year	Chr.	LD block region (bp)	Peak position (bp)	–log_10_(*p*)	Major/minor allele*^a^*	Homozygote for major allele		Homozygote for minor allele		MAF*^b^*	Fixed effect at SNP	Known locus
						No. of Acc.	Ave. DTH	No. of Acc.	Ave. DTH			
2019	3	31,006,150–31,311,258	31,063,052	9.74	G/A	135	95.89	116	114.42	0.461	6.11	*PhyC*
2020	3	31,006,150–31,311,258	31,115,122	9.45	C/T	134	95.96	117	113.75	0.464	5.80	*PhyC*
Two years	3	31,006,150–31,311,258	31,063,052	9.21	G/A	135	95.55	116	113.53	0.462	5.78	*PhyC*

*^a^* Reference and alternative alleles based on Os-Nipponbare-Reference-IRGSP-1.0. *^b^* Minor allele frequency

**Table 2.** QTL conferring days to heading in the biparental F_2_ population

QTL	Chr.	QTL region*^a^*		Peak marker*^a^*	Peak LOD	PVE*^b^* (%)	Additive effect*^c^*	Dominance effect	LOD threshold*^d^*	GWAS peak	Overlapping with known loci
*qDTH3*	3	30,351,246	31,694,280	30,809,923	6.39**	22.08	2.74	–1.42	4.18	MTA3	*PHYC*, *Hd6*
*qDTH6.1*	6	2,940,130	3,563,230	2,940,130	6.29**	21.76	3.19	–1.51	4.18		*RFT1*, *Hd3a*
*qDTH6.2*	6	5,660,320	7,843,157	7,206,244	4.81*	17.12	2.27	–2.51	4.18		
*qDTH8*	8	3,317,965	5,572,424	4,020,088	8.09**	27.08	2.96	2.04	4.18		*DTH8/Ghd8/Hd5*

** significant at 1% level *^a^* The physical position was estimated based on Os-Nipponbare-Reference-IRGSP-1.0. *^b^* Percentage of variance explained *^c^* Genetic effect of CC319 *^d^* LOD threshold at 5% significant level

**Table 3.** Number of accessions harboring PHYCHapA and PHYHapC haplotypes by the seven geographical regions in Myanmar

Region	PHYCHapA (A)	PHYCHapC (C)	Ratio (A : C)	*χ* ^2^_73:84_
EMR	10	30	13:45	13.52***
WMR	2	6		
NMR	1	9		
CDZ	17	22	17:22	0.13
WCR	7	3	42:15	16.94***
ECR	12	5		
DR	23	7		
NA	1	2		
Total	73	84		
