# Supplementary material for: A genome-wide association study using Myanmar indica diversity panel reveals a significant genomic region associated with heading date in rice
Source: Breed Sci. 2024 Dec 4;74(5):415–26. doi: 10.1270/jsbbs.23083 (PMC11780332; doi:10.1270/jsbbs.23083)
Supplement: Supplementary file 1 — Supplemental Figures [file 74_415_s1.pdf]

doi: 10.1270/jsbbs.23083

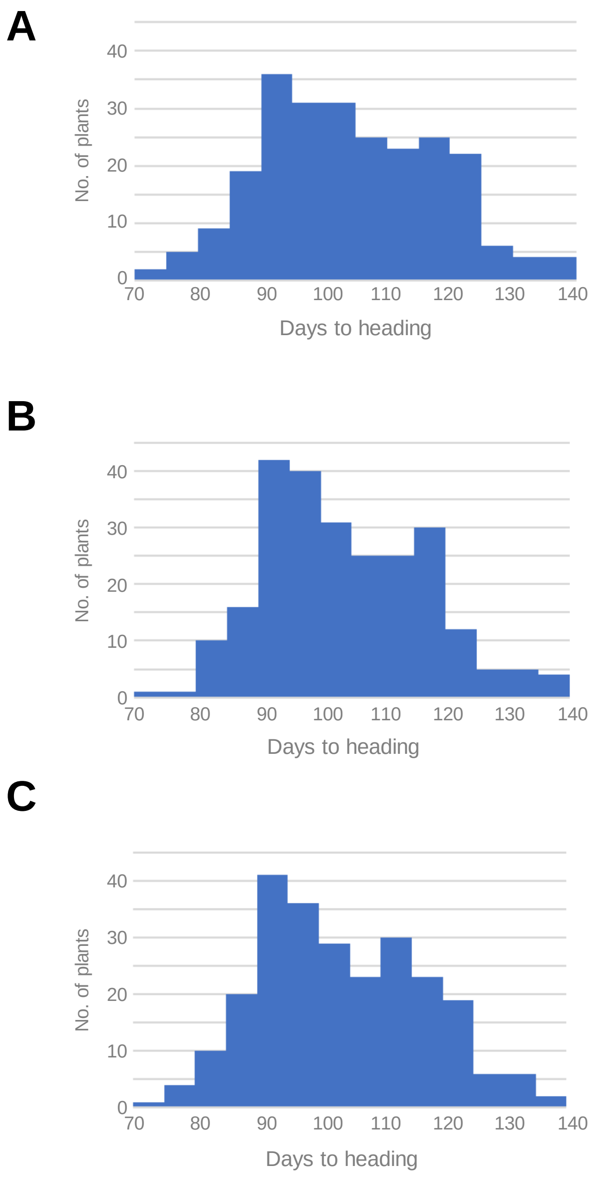


**Supplemental Fig. 1. Frequency distribution of days to heading in the MIDP grown on monsoon season at Nay Pyi Taw, Myanmar.** (**A-C**) Frequency distributions of days to heading on monsoon seasons in 2019 (**A**), 2020 (**B**), and the two-year mean (**C**).

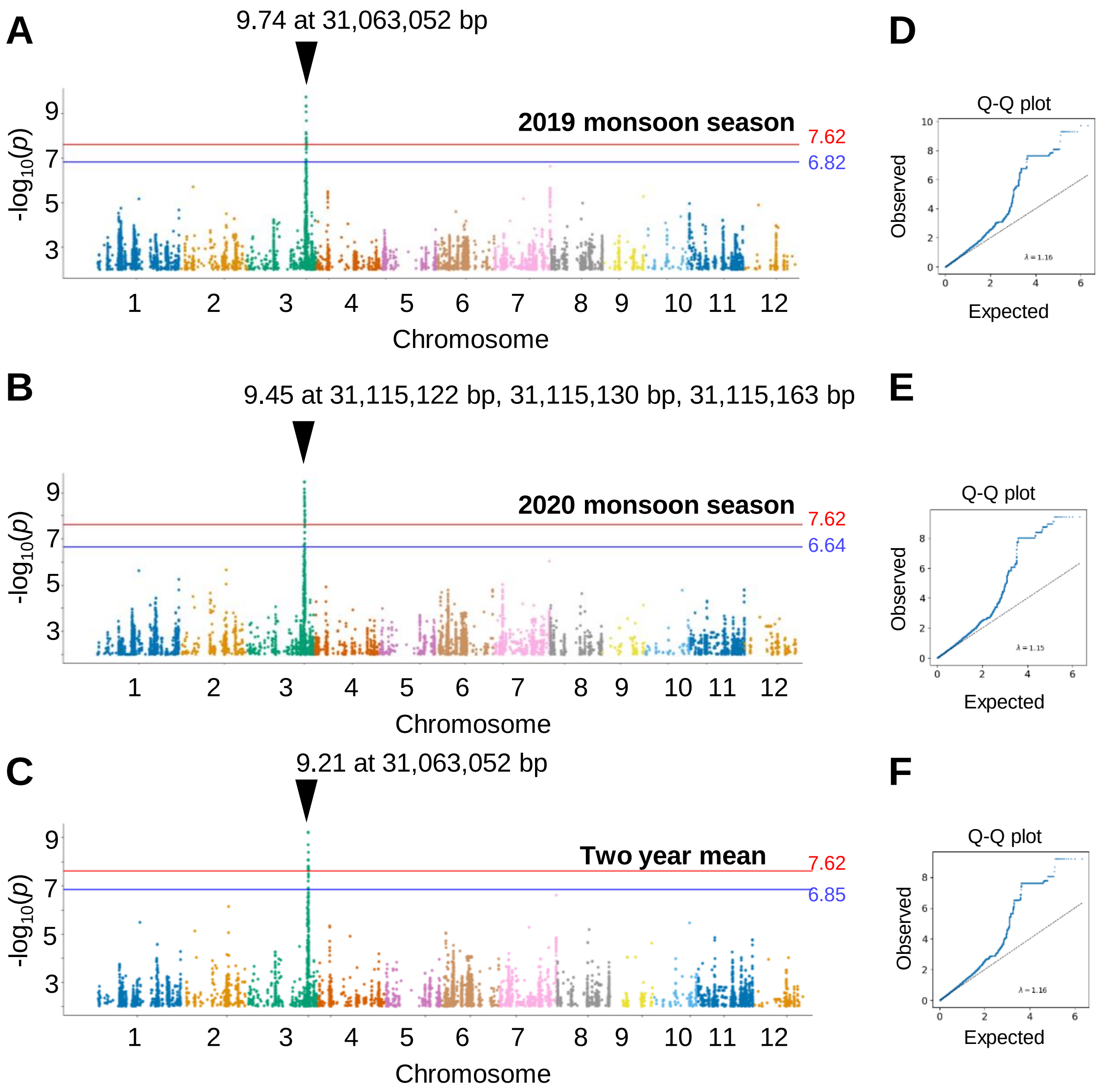


**Supplemental Fig. 2. Genome-wide association of days-to-heading in the MIDP grown during monsoon season of Myanmar by permutation-based GWAS (permGWAS).** (**A-C**) Manhattan plots of GWAS in monsoon season in 2019 (**A**), 2020 (**B**), and the two-year mean (**C**). The red and blue lines indicate thresholds at 5% significance level determined by Bonferroni correction and 100 times permutation test, respectively. (**D-F**) Quantile-quantile plot of observed and expected -$\log_{10}(p)$ in 2019 (**D**), 2020 (**E**), and the two-year mean (**F**).

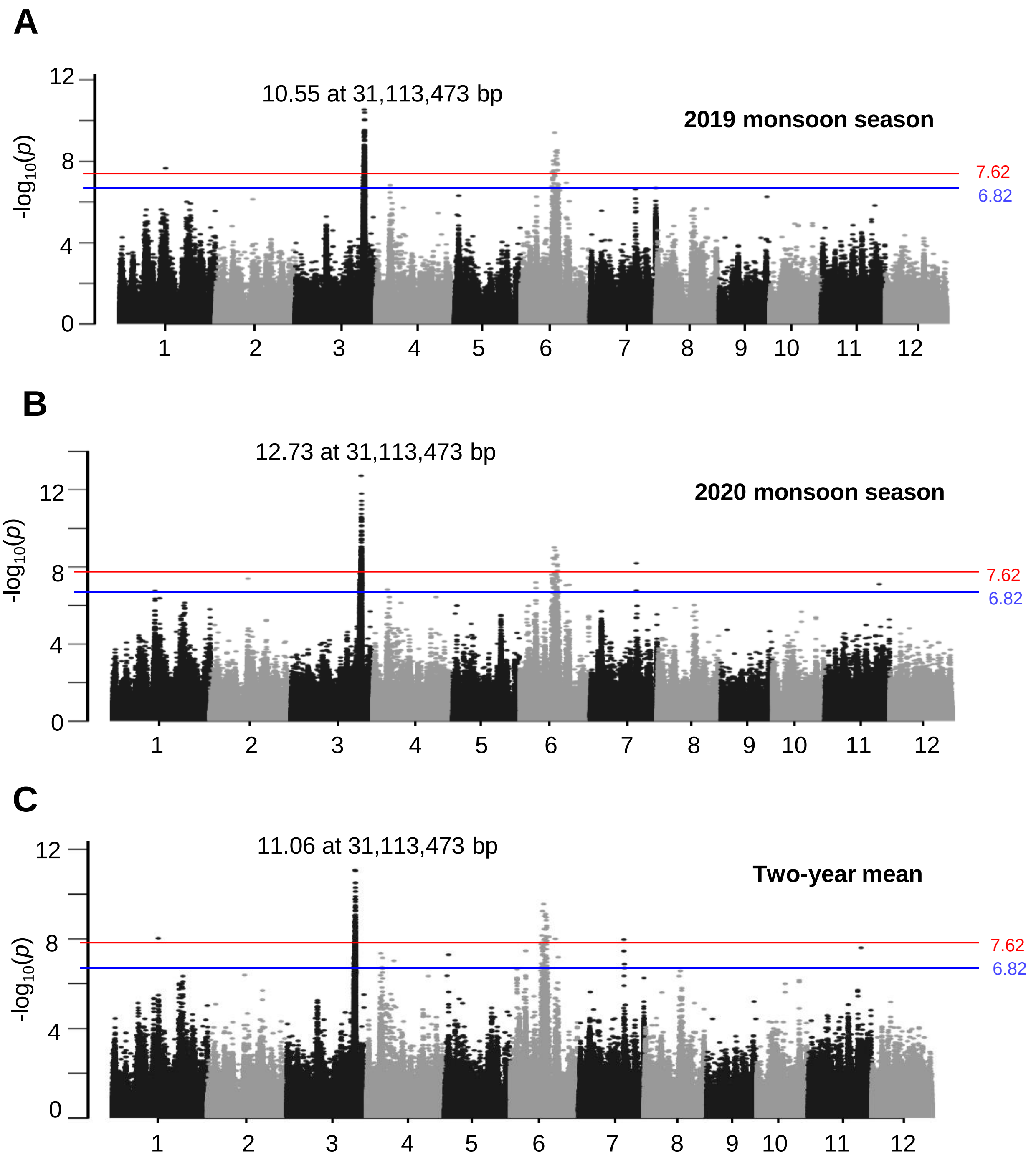


**Supplemental Fig. 3. Genome-wide association of days to heading in the MIDP grown during monsoon season of Myanmar by genomic best linear unbiased prediction (gBLUP).** (**A-C**) Manhattan plots of GWAS in monsoon season in 2019 (**A**), 2020 (**B**), and the two-year mean (**C**). The red and blue lines indicate thresholds at 5% significance level determined by Bonferroni correction and 100 times permutation test, respectively.

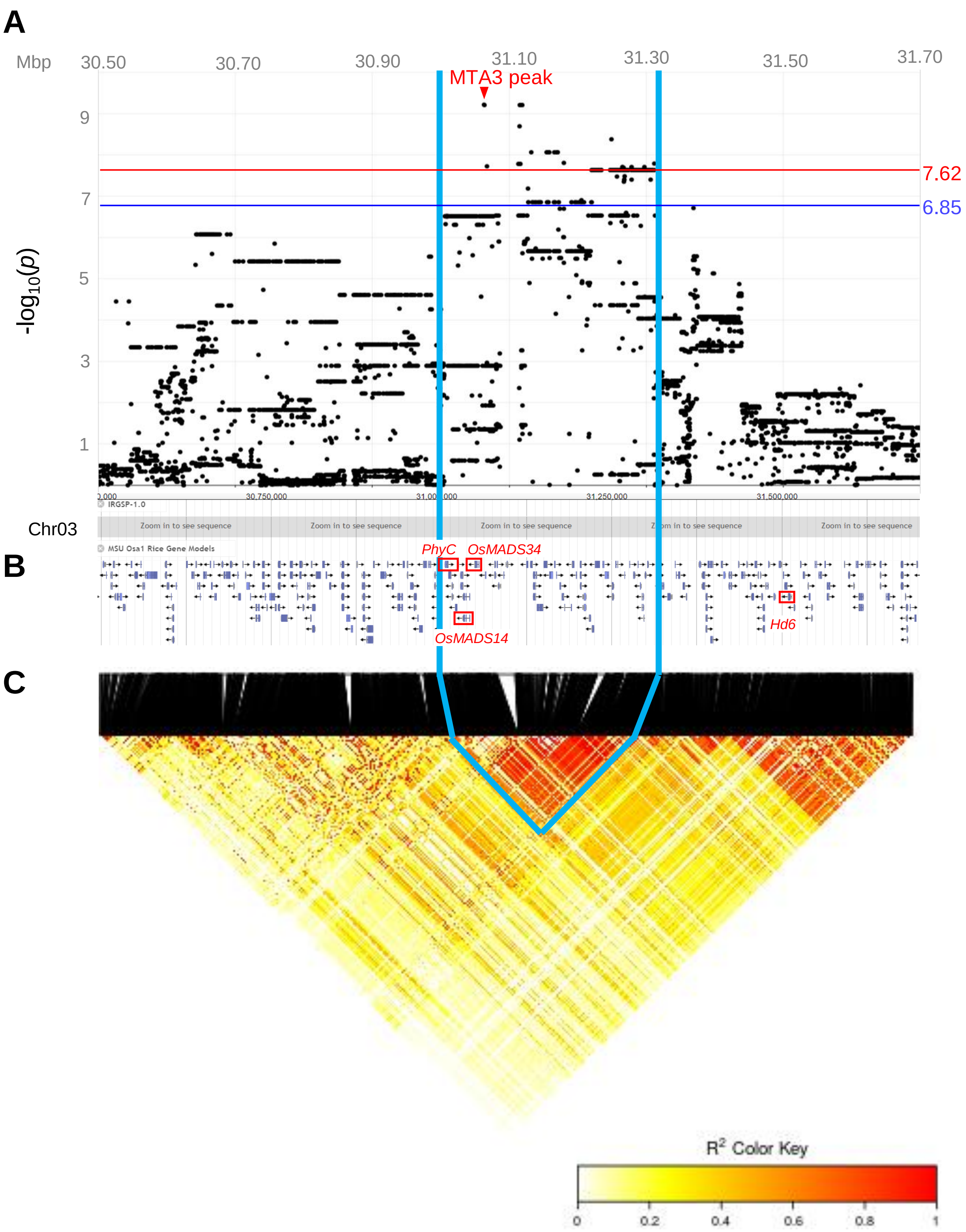


**Supplemental Fig. 4. Association and linkage disequilibrium plot of the MTA3 region and candidate region on the chromosome.** (**A**) Local Manhattan plot and candidate genes in the MTA3 region for two-year mean of days to heading in 2019 and 2020 monsoon seasons. Blue vertical lines represent the LD block containing the peak SNP of MTA3, shown as a red dot. (**B**) Annotation of genes included in the MTA3 region. The genes in the red rectangles represent possible candidate genes for MTA3 peak. (**C**) LD heatmap around the MTA3 region.

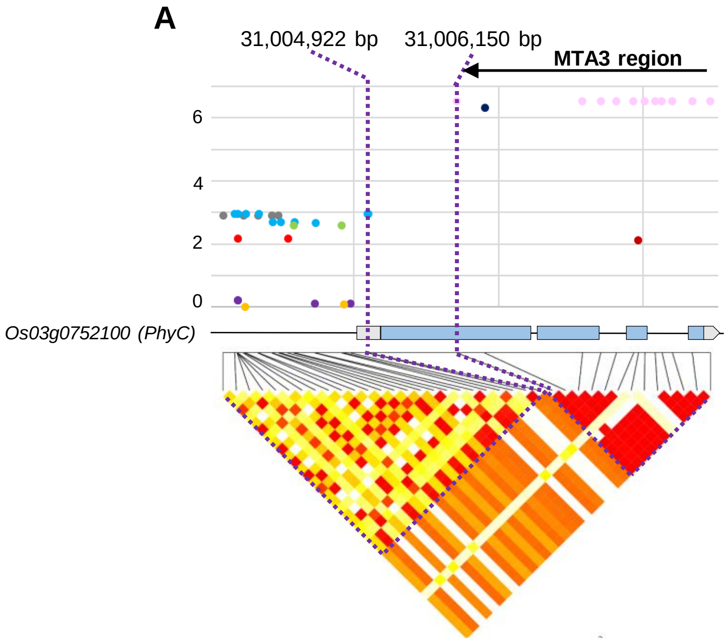


**B**

| | Segregation pattern | | | | | | | | |
|---|---|---|---|---|---|---|---|---|---|
| | a | b | c | d | e | f | g | h | i |
| Ref | 0 | 0 | 0 | 0 | 0 | 0 | 0 | 0 | 0 |
| Alt | 1 | 1 | 1 | 1 | 1 | 1 | 1 | 1 | 1 |
| HapA | 0 | 0 | 0 | 0 | 0 | 1 | 0 | 1 | 0 |
| HapB | 0 | 1 | 1 | 0 | 1 | 0 | 0 | 1 | 0 |
| HapC | 1 | 1 | 0 | 1 | 0 | 0 | 1 | 0 | 0 |
| HapD | 0 | 1 | 1 | 0 | 1 | 0 | 1 | 0 | 0 |
| HapE | 0 | 1 | 0 | 1 | 1 | 0 | 1 | 0 | 0 |
| HapF | 0 | 1 | 1 | 0 | 1 | 0 | 1 | 0 | 1 |

**Supplemental Fig. 5. Association and linkage disequilibrium plot of the *PHYC* region on chromosome 3.** (**A**) Local Manhattan plot of the *PhyC* gene, including the 2 kb promoter region. (**B**) Table in the right side shows segregation pattern in the *PhyC* gene and the 2 kb promoter region.

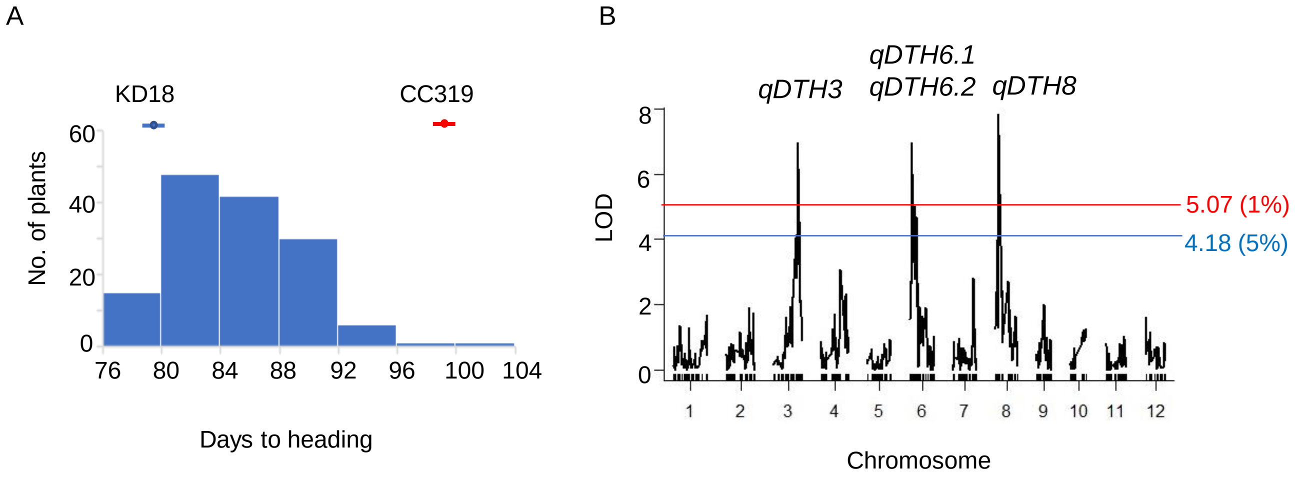


**Supplemental Fig. 6. Biparental QTL analysis in an $F_2$ population derived from the Myanmar accession CC319 and the Vietnamese cultivar KD18**.
(**A**) Frequency distribution of DTH in the $F_2$ population. n = 118. (**B**) Genome-wide distribution of logarithm of odds (LOD) obtained by marker regression analysis. The red and blue lines indicate significance thresholds at the 1% and 5% levels, respectively,
